# Recurrence and survival of patients with stage III endometrial cancer after radical surgery followed by adjuvant chemo- or chemoradiotherapy: a systematic review and meta-analysis

**DOI:** 10.1186/s12885-022-10482-x

**Published:** 2023-01-09

**Authors:** Si-yu Cao, Yu Fan, Yu-fei Zhang, Jia-ying Ruan, Yi Mu, Jin-ke Li

**Affiliations:** 1 Department of Gynecology and Obstetrics, West China Second Hospital, Sichuan University, No. 20, Section 3, Renminnan Road, Chengdu, 610041 Sichuan China; 2 Key Laboratory of Birth Defects and Related Diseases of Women and Children, Ministry of Education, Sichuan University, Chengdu, People’s Republic of China

**Keywords:** Endometrial cancer, Chemotherapy, Radiotherapy, Chemoradiotherapy, Meta-analysis

## Abstract

**Objective:**

To compare recurrence and survival in patients with stage III endometrial cancer after radical surgery, followed by either adjuvant chemoradiotherapy (ACR) or adjuvant chemotherapy (AC).

**Methods:**

We searched for relevant studies in PubMed Central, Embase and the Cochrane Central Register of Controlled Trials. Data were pooled on rates of recurrence as well as rates of progression-free, disease-free and overall survival. Heterogeneity was evaluated using the I^2^ test. Subgroup and sensitivity analyses were performed to identify potential sources of heterogeneity.

**Results:**

Data from 18,375 patients in 15 retrospective studies and one randomized controlled trial were meta-analyzed. Compared to the AC group, the ACR showed significantly lower risk of local recurrence (OR 0.43, 95%CI 0.32–0.59) and total recurrence (OR 0.72, 95%CI 0.58–0.89). ACR was also associated with significantly better overall survival (HR 0.66, 95%CI 0.57–0.76), progression-free survival (HR 0.56, 95%CI 0.39–0.81) and disease-free survival (HR 0.66, 95%CI 0.53–0.83).

**Conclusions:**

Adding adjuvant radiotherapy to adjuvant chemotherapy after radical surgery may significantly reduce risk of local and overall recurrence, while significantly improving survival of patients with stage III endometrial cancer.

## Introduction

Endometrial cancer is one of the most frequent malignancies in the female reproductive system, and the morbidity and mortality associated with the disease continue to rise [1, 2], including in developed countries [3]. In 2021 alone, 604,127 new cases and 341,831 deaths were reported around the world [4]. Rates of 5-year overall survival (OS) with endometrial cancer can be as high as 80%, making prognosis better than with ovarian or cervical cancer [1]. However, the rate of 5-year OS is lower than 20% for patients with advanced or recurrent endometrial cancer [5].

Based on the International Federation of Gynecology and Obstetrics (FIGO) staging system [6], the most widely used in endometrial cancer, stage III endometrial cancer can involve the uterine serosa and/or adnexa (substage IIIA), vagina or parametrium (IIIB), or pelvic or para-aorta lymph node (IIIC). The usual therapy for stage III patients is radical surgery involving total hysterectomy and bilateral salpingo-oophorectomy (TH/BSO), sometimes together with pelvic or para-aortic lymph node dissection [7, 8].

The National Comprehensive Cancer Network (NCCN) recommends adjuvant therapies after radical surgery, which can involve chemo- and radiotherapy, on their own or combined [8]. On its own, adjuvant chemotherapy (AC) can significantly improve survival [9], but it does not appear to decrease the rate of distant recurrence. On its own, radiotherapy of the pelvic area or entire abdominal area can reduce the rate of pelvic recurrence but not the rate of distant recurrence, limiting long-term survival benefit [10–12].

Combining the two therapies into adjuvant chemoradiotherapy (ACR) may make up for the shortcomings of each therapy on its own [13]. However, whether ACR is associated with better prognosis than AC is unclear: some studies have reported significantly better progression-free and overall survival with ACR [14, 15], whereas others have found no difference between AC and ACR in recurrence-free survival among patients with endometrial cancer in stages III-IVA [12, 16, 17]. Large randomized trials are currently comparing ACR and AC for patients with stage III disease [18, 19], but the data are not yet sufficiently mature for analysis.

Therefore we undertook the present study to assess the available evidence on whether ACR provides benefits over AC in the treatment of stage III endometrial cancer patients after primary radical surgery.

## Materials and method

This meta-analysis was performed in strict accordance with the Preferred Reporting Items for Systematic Reviews and Meta-analyses (PRISMA) statement, and it was registered with the International Prospective Register of Systematic Reviews (CRD42022312042).

### Literature search strategy

The databases in PubMed Central, Embase and Cochrane Central Register of Controlled Trials (CENTRAL) were searched to identify relevant randomized controlled trials (RCTs), case control trials and cohort trials published between January 1, 2012 and March 1, 2022. Two authors (SY Cao and Y Fan) independently searched all databases. The following search string was used in all databases: *(endometrial neoplasm OR endometrial cancer OR endometrial carcinoma OR endometrium carcinoma OR endometrium neoplasm OR endometrium neoplasm) AND (postoperative OR hysterectomy OR surgical) AND (chemotherapy, adjuvant OR pharmacotherapy OR radiotherapy, adjuvant)*.

We also manually screened the reference lists of articles to identify additional potentially eligible studies. If multiple studies appeared to analyze overlapping patient populations, we included only the most recent one. If overlapping studies had the same publication year, we included only the one reporting the most detailed, complete analyses.

### Study selection

To be included in the review and meta-analysis, studies had to satisfy the following inclusion criteria: (1) patients were diagnosed with endometrial carcinoma and definitively assigned to FIGO stage III (regardless of whether substage was A, B, or C) based on histopathology or imaging; (2) patients underwent primary surgery involving TH/BSO; (3) patients received AC or ACR after surgery; (3) the study design was randomized-controlled, observational prospective cohort, retrospective cohort or case–control; (4) relevant outcomes were reported, such as rates of recurrence, progression-free survival (PFS), disease-free survival (DFS) and overall survival (OS); and (5) the full text of the study was available.

Studies were excluded if they (1) were not original research articles, such as reviews, study protocols, comments or letters; (2) were not published in English; (3) involved patients who received neoadjuvant therapy before surgery; (4) did not report necessary data; or (5) failed to score adequately in the quality assessment (see Section 2.3).

### Quality assessment

Two researchers (SY Cao and Y Fan) independently assessed the methodological quality of the included studies. We used the Newcastle–Ottawa Quality Assessment Scale (NOS) [20] to evaluate the quality of non-randomized studies, and the Jadad/Oxford quality scoring system [21] to evaluate the quality of RCTs. Only non-randomized studies with total scores of at least five points or RCTs with total scores of at least three points were included in the final meta-analysis. Any disagreements were resolved by discussion with the corresponding author.

### Data extraction

Two authors (SY Cao and Y Fan) independently extracted data from included studies. Extracted data included: first author, country or region, FIGO stage and substage, age of patients, year of publication, study name, number of patients enrolled, study type, type of AC, type of ACR, rate of recurrence (local, distant and total), PFS, DFS, OS, types of primary surgery and median length of follow-up. Any disagreements were resolved by discussion with the corresponding author.

Patients were defined to have experienced recurrence if they initially achieved complete remission after AC or ACR, but subsequently suffered recurrent cancer anywhere in the body, based on histopathology or imaging [8]. Relapse in the vaginal vault, vagina, pelvic cavity, pelvic and/or para-aortic nodes was considered local recurrence [17]; relapse at other sites was defined as distant recurrence [16]. OS, DFS and PFS were extracted directly from the studies or from the published Kaplan–Meier plots. Any uncertainties about data extraction and classification were resolved by discussion with the corresponding author.

### Data analysis

Statistical analyses were calculated using the *metan*, *metabias* and *metaprop* packages in STATA 16.0 (Statacorp, College Station, TX, USA). We calculated odds ratios (ORs) or hazard ratios (HRs) and the associated 95% confidence intervals (CIs). When the included studies did not directly report HR for OS or PFS, the necessary data were extracted using Engauge Digitizer 4.1 (http://sourceforge.net/projects/digitizer/).

Meta-analysis was initially conducted using a random-effects model, and heterogeneity was assessed in terms of I^2^, where I ^2^ ≥ 50% was considered high heterogeneity [22]. Potential sources of heterogeneity were explored by performing subgroup analysis based on FIGO substage (IIIA, IIIB or IIIC) or histological type (endometrioid or non-endometrioid carcinoma), and by repeating the meta-analysis after removing one study at a time. We stratified patients into endometrioid or non-endometrioid types because non-endometrioid carcinomas are less common and quite heterogeneous in histopathology, comprising carcinosarcoma, serous adenocarcinoma, clear cell adenocarcinoma, and other rare types [1]. Publication bias was assessed using Begg's test [23].

## Results

### Characteristics of included studies

**Fig. 1:**
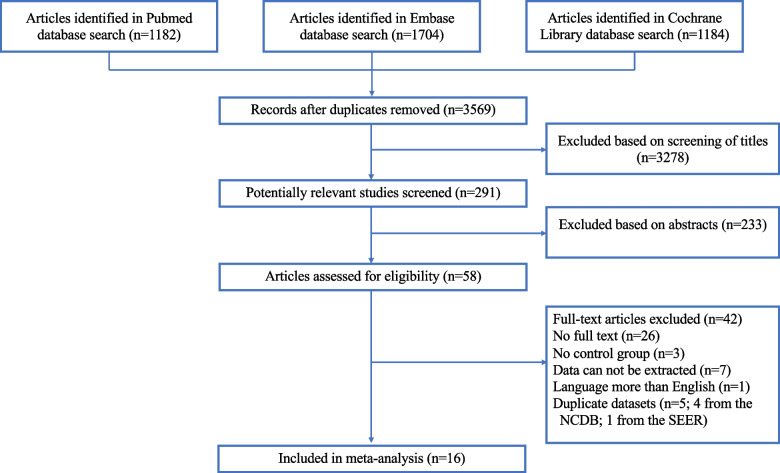
Flow diagram of study selection. Abbreviations: NCDB, National Cancer Database; SEER, Surveillance, Epidemiology, and End Results Database

The results of the literature search and screening can be found in Fig. 1. A total of 3,569 articles were searched, which after careful screening led to a final set of 16 articles with 18,375 patients [14–17, 24–35], of which 15 were retrospective cohort studies and one was an RCT. The studies were conducted in the United States (9), Italy (2), Taiwan (2), United Kingdom (1), Netherlands (1), and in regions of eastern Europe and central Asia (1). The characteristics and quality scores of the included articles are listed in Table 1.

**Table 1:** Characteristics of included studies

Study	Country or area	Design	Stage (% of patients)	no. of patients	Median age (years)	Adjuvant therapy (n)	Adjuvant regimen	Histology (% of patients)	Median follow-up (months)	Type of primary surgery (% of patients)	Quality assessment^a^
McEachron 2021 [14]	USA	R	IIIC (100%)	144	NR	AC (40)	TP; PTC; other	E (17%)	66	TH/BSO + pelvic/para aortic lymph node dissection (100%)	7
						ACR (104)	EBRT ± VBT	NE: SC (37%); CS (42%); CCA (4%)			
Montes de Oca 2021 [17]	USA	R	IIIA (100%)	671	58.3	AC (286)	NR	E (100%)	NR	TH/BSO only (22.7%)	6
						ACR (385)	EBRT, VBT			TH/BSO + pelvic/para aortic lymph node dissection (77.3%)	
Bogani 2020 [27]	USA	R	IIIC (100%)	80	NR	AC (24)	TC; TP; ACP; CP; PTC	E (48%)	NR	TH/BSO + pelvic/para aortic lymph node dissection (100%)	7
						ACR (56)	EBRT	NE (52%)			
Huang 2020 [31]	Taiwan	R	IIIA (32%), IIIB (6%), IIIC (62%)	125	57	AC (67)	TP; TC; AP	E: 84%	44	TH/BSO + pelvic lymph node dissection (100%)	8
						ACR (58)	EBRT, VBT	NE: 16%			
Ko 2020 [28]	USA	R	IIIA (41%), IIIB (17%), IIIC (42%)	1301	73	AC (779)	TP	E (63%)	43.2	TH/BSO only (9.2%)	7
						ACR (522)	EBRT, VBT	NE: SC (21%); CCA (4%); CS (12%)		TH/BSO + pelvic/para aortic lymph node dissection (80.8%)	
Lee 2020 [32]	Taiwan	R	IIIC (100%)	441	55	AC (142)	NR	E (82%)	43.2	TH/BSO + pelvic/para aortic lymph node dissection (100%)	7
					57	ACR (299)	EBRT	NE (18%)			
van Weelden 2020 [25]	Netherlands	R	IIIA (30%), IIIB (8%), IIIC (62%)	333	65.8	AC (158)	NR	E (36%)	NR	TH/BSO only (25.5%)	6
					62.9	ACR (175)	EBRT	NE: SC (40%); CCA (5%); CS (19%)		TH/BSO + pelvic/para aortic lymph node dissection (74.5%)	
Verrengia 2020 [34]	Italy	R	IIIA (28%), IIIB (9%), IIIC (63%)	32	NR	AC (14)	TP	E (72%)	31	TH/BSO + pelvic/para aortic lymph node dissection (100%)	6
						ACR (18)	EBRT, VBT	NE (28%)			
Kahramanoglu 2019 [16]	Central Asia/Eastern Europe	R	IIIA (32%), IIIB (2%), IIIC (66%)	787	NR	AC (242)	TC	E (100%)	42	TH/BSO only (15.6%)	6
						ACR (545)	EBRT ± VBT			TH/BSO + pelvic/para aortic lymph node dissection (84.4%)	
Kidd 2019 [15]	USA	R	IIIA (23%), IIIB (5%), IIIC (72%)	13,072	60	AC (7847)	NR	E (100%)	44.4	TH/BSO only (24.8%)	7
					60	ACR (5225)	EBRT			TH/BSO + pelvic/para aortic lymph node dissection (75.2%)	
Matei 2019 [12]	USA	RCT	IIIA (21%), IIIB (3%), IIIC (73%), IVA (1%)	736	60	AC (366)	TP	E (70%)	47	TH/BSO + pelvic and paraaortic lymph node biopsy or dissection (100%)	5
						ACR (370)	EBRT	NE: SC (18%); CCA (3%); other (9%)			
Albuquerque 2018 [24]	USA	R	IIIA (39%), IIIB (8%), IIIC (53%)	181	62	AC (57)	TP; ACP; PTC	E (66%)	31.5	TH/BSO + pelvic/para aortic lymph node dissection (100%)	5
						ACR (124)	EBRT; VBT	NE (34%)			
Binder 2017 [35]	USA	R	IIIC (100%)	146	67.5	AC (46)	TP; AP	E (73%)	40.1	TH/BSO + pelvic/para aortic lymph node dissection (100%)	8
					60.5	ACR (100)	EBRT, IMRT, VBT	NE: CCA(14%); other (23%)			
Signorelli 2015 [26]	Italy	R	IIIA (45%), IIIB (7%), IIIC (48%)	75	61	AC (46)	ACP; PAC; CP; CT; other	E/NE (NR)	101	TH/BSO only (23%)	6
						ACR (29)	EBRT			TH/BSO + pelvic/para aortic lymph node dissection (77%)	
Kuku 2013 [29]	UK	R	IIIA (60%), IIIB (10%), IIIC (30%)	58	67	AC (7)	TP	E (65%)	58	TH/BSO only (70%)	7
						ACR (51)	EBRT ± VBT	NE: PSC (16%); CCA (4.3%); MCC (5.3%); other (9.4%)		TH/BSO + selective pelvic and/ or para-aortic lymph node dissection (30%)	
Secord 2013 [30]	USA	R	IIIC (100%)	207	61	AC (46)	TP; ACP; PTC; CT; other	E (66%)	42	TH/BSO + selective or systematic lymphadenectomy (pelvic or pelvic and/or aortic) (100%)	6
					60	ACR (161)	WPRT; WAR; VBT	NE: PSC (11%); MCC (18%); other (5%)			

*R* Retrospective, *E* Endometroid, *NE* Non-endometroid, *AC* Adjuvant chemotherapy, *ACR* Chemoradiotherapy, *N* Number of all patients, *CCA* Clear cell carcinoma, *CS* Carcinosarcoma, *PSC* Papillary serous carcinoma, *SC* Serous carcinoma, *MCC* Mixed-cell carcinoma, *NR* Unknown, *T* Taxol, *A* Adriamycin, *P* Platinum, *TC* Taxol + cisplatin, *TP* Taxol + carboplatin, *PC* Carboplatin + cyclophosphamide, *ACP* Adriamycin + cisplatin, *AP* Adriamycin + carboplatin, *PAC* Adriamycin + cyclophosphamide + cisplatin, *CP* Cyclophosphamide + cisplatin, *PTC* Platinum + Taxol + Adriamycin, *CT* Taxol only, *EBRT* External pelvic radiotherapy, *VBT* Vaginal brachytherapy, *IMRT* Image-guidance or intensity-modulated radiation therapy, *WAR* Whole abdominal radiotherapy, *WPRT* Whole pelvic radiotherapy, *TH/BSO* Total hysterectomy and bilateral salpingo-oophorectomy

^a^Cohort studies were measured by the Newcastle–Ottawa Quality Assessment Scale (NOS) and RCT was measured by Jadad/Oxford

### Quality assessment

Table 2 shows the quality assessment of retrospective studies. The quality of the RCT was assessed based on five scores

**Table 2:** Quality of retrospective studies, as assessed on the Newcastle–Ottawa Quality Assessment Scale (NOS)

Study	Selection				Comparability	Outcomes			Total score
	Definition of exposed cohort	Selecting non-exposed cohort	Ascertainment of exposure	Outcomes was not present at start	Comparing cohorts of design and analysis	Assess outcome	Follow-up time (> 3 years)	Adequacy of follow-up of cohorts	
McEachron (2021) [14]	★	★	★		★★	★	★		7
Montes de Oca (2021) [17]	★	★	★		★★	★			6
Bogani (2020) [27]	★	★	★		★★	★		★	7
Huang (2020) [31]	★	★	★		★★	★	★	★	8
Ko (2020) [28]	★	★	★		★★	★	★		7
Lee (2020) [32]	★	★	★		★★	★	★		7
van Weelden (2020) [25]	★	★	★		★★	★			6
Verrengia (2020) [34]	★	★	★		★	★		★	6
Kahramanoglu (2019) [16]	★	★	★		★	★	★		6
Kidd (2019) [15]	★	★	★		★★	★	★		7
Albuquerque (2018) [24]	★	★	★		★	★			5
Binder (2017) [35]	★	★	★		★★	★	★	★	8
Signorelli (2015) [26]	★	★	★		★	★	★		6
Kuku (2013) [29]	★	★	★		★	★	★	★	7
Secord (2013) [30]	★	★	★		★	★	★		6

### Recurrence rate

**Fig. 2:**
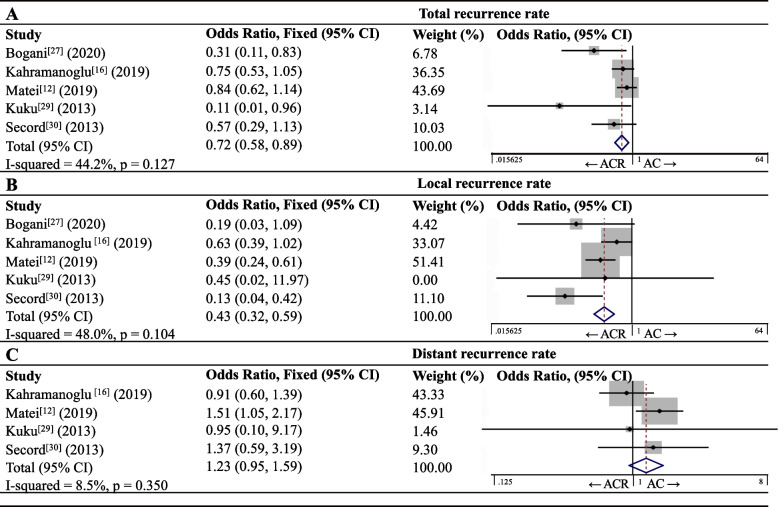
Forest plots of rates of **A** total recurrence, **B** local recurrence or **C** distant recurrence in patients with endometrial carcinoma after adjuvant therapy

Five articles [12, 16, 27, 29, 30] involving 1,868 patients reported recurrence rates for AC and ACR groups (Fig. 2A). Meta-analysis showed a total recurrence rate of 34.9% (239/685) for the AC group and 27.6% (326/1183) for the ACR group, corresponding to significantly lower risk of recurrence with ACR: OR 0.72, 95%CI 0.58–0.89 (I^2^ = 44.2%, *p* = 0.127). Similarly, the rate of local recurrence was significantly lower in the ACR group (7.2% vs 16.5%, OR 0.43, 95%CI 0.32–0.59; I^2^ = 48.0%, *p* = 0.104; Fig. 2B). In contrast, distant recurrence rate was similar between the ACR and AC groups (18.5% vs 16.5%, OR 1.23, 95%CI 0.95–1.59; I^2^ = 8.5%, *p* = 0.350; Fig. 2C).

### OS rate

**Fig. 3:**
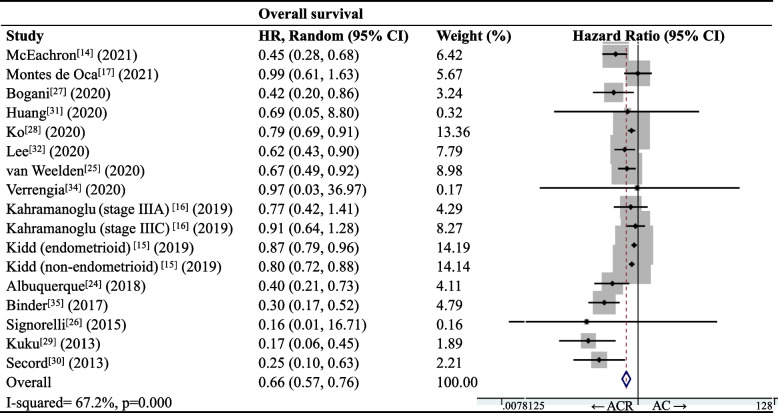
Forest plot of overall survival of patients with endometrial carcinoma after adjuvant therapy

Meta-analysis of data for 17,639 patients in 15 studies [14–17, 24–32, 34, 35] showed that ACR was associated with significantly better OS than AC (HR 0.66, 95%CI 0.57–0.76; I^2^ = 67.2%, *p* < 0.001; Fig. 3).

Given the high heterogeneity of the pooled data, we conducted two subgroup analyses.

**Fig. 4:**
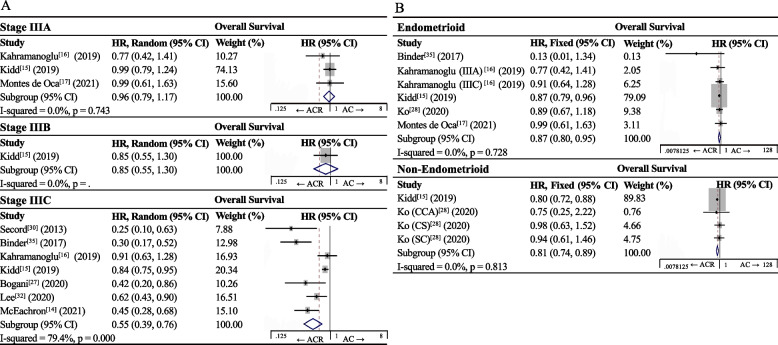
Forest plot of overall survival of patients with endometrial carcinoma after adjuvant therapy, stratified by **A** FIGO stage or **B** histology type

The significantly better OS with ACR than AC was observed in the subgroup of stage IIIC patients (HR 0.55, 95%CI 0.39–0.76, I^2^ = 79.4%, *p* < 0.001) [14–17, 27, 30, 32, 35], but not in the subgroup of IIIA patients (HR 0.96, 95%CI 0.79–1.17; I^2^ = 0.0%, *p* = 0.743) [15–17] (Fig. 4A). We could not calculate an HR for the subgroup of IIIB patients since only one study reported relevant data [15].

The significant OS benefit of ACR over AC was observed separately in the subgroup of patients with endometrioid carcinoma (HR 0.87, 95%CI 0.80–0.95; I^2^ = 0.0%, *p* = 0.728) and in the subgroup with non-endometrioid carcinoma (HR 0.81, 95%CI 0.74–0.89; I^2^ = 0.0%, *p* = 0.744; Fig. 4B).

Repeating the meta-analysis after removing each study one by one did not significantly change the original results, so we were unable to identify obvious sources of heterogeneity.

### Rates of PFS and DFS

**Fig. 5:**
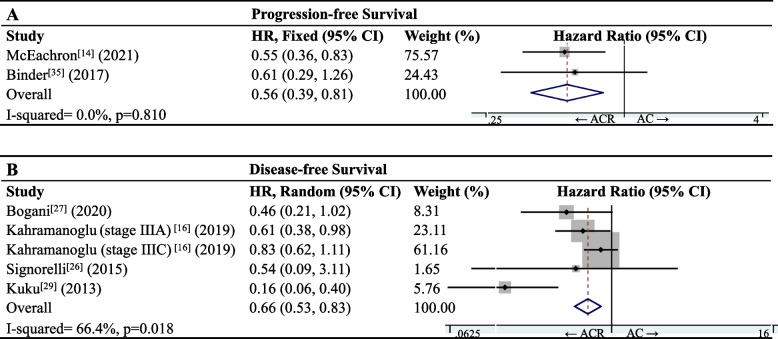
Forest plot of **A** progression-free or **B** disease-free survival of patients with endometrial carcinoma after adjuvant therapy

Meta-analysis of PFS data for 290 patients in two studies [14, 35] showed that ACR was associated with significantly better PFS than AC (HR 0.56, 95%CI 0.39–0.81; I^2^ = 0.0%, *p* = 0.810; Fig. 5A). Similarly, meta-analysis of data for 1,000 patients in four studies [16, 26, 27, 29] showed that ACR was associated with significantly better DFS (HR 0.66, 95%CI 0.53–0.83; I^2^ = 66.4%, *p* = 0.018; Fig. 5B).

### Publication bias

**Fig. 6:**
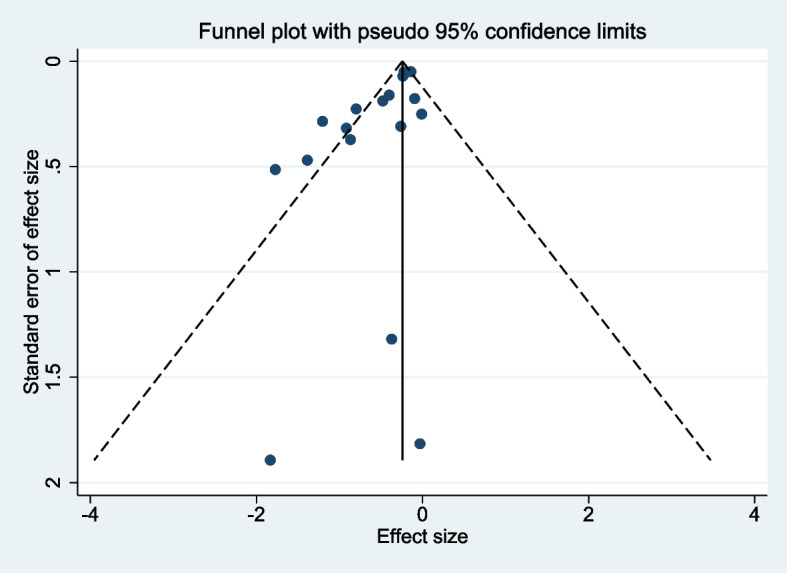
Funnel plot of hazard ratios for overall survival reported in 15 studies

Publication bias was calculated for the meta-analysis of OS, for which most studies reported data [14–17, 24–32, 34, 35]. Begg’s test suggested no potential publication bias (*p* = 0.108), and the funnel chart showed a symmetric distribution (Fig. 6).

## Discussion

In this meta-analysis of patients with FIGO stage III endometrial cancer who underwent radical surgery, we compared cancer recurrence rates and survival between those who received AC or ACR after surgery. The two adjuvant treatments were associated with similar distant recurrence, while ACR was associated with significantly lower risk of total recurrence and local recurrence. These meta-analyzed outcomes were associated with low heterogeneity. ACR was also associated with significantly higher OS than AC, but this outcome was associated with high heterogeneity, whose source we failed to identify.

Our work confirms the results of a recent, smaller meta-analysis comparing AC and ACR on survival of patients with endometrial carcinoma [36]. Our work extends that previous analysis by comparing, for the first time, rates of local and distant recurrence as well as PFS and DFS. The results from our meta-analysis are also consistent with several studies that, drawing on the US National Cancer Database, suggest better survival with ACR than AC [18, 37, 38]. In addition, we found evidence of better survival with ACR than AC specifically in patients with endometrial cancer in stage IIIC. These results suggest that ACR is more effective at preventing cancer recurrence and improving OS in the presence of pelvic lymph node involvement (substage IIIC1) or para-aortic lymph node metastasis (substage IIIC2). ACR may provide greater benefit than AC to patients in stage IIIC because such patients are at higher risk of local or distant recurrence. In contrast, ACR may not offer greater benefit than AC to patients in stage IIIA because such patients do not have distant metastasis or lymph node involvement.

We found evidence of better survival with ACR than AC in subgroups of patients with endometrioid or non-endometrioid carcinoma. This is an important finding because non-endometrioid carcinomas include severe types such as carcinosarcoma, serous carcinoma and clear cell adenocarcinoma, which are associated with greater risk of recurrence and worse survival. In fact, our finding is consistent with reports that ACR provides greater overall survival benefit than AC to patients with serous carcinoma [39, 40]. This literature and our meta-analysis suggest that ACR may lead to better prognosis in patients with severe or advanced endometrial cancer.

Our findings differ from those in the large RCT “GOG-258” [12], but that RCT may underestimate the ability of ACR to reduce recurrence rate because of an insufficient radiation dose in the combination regime [12, 18]. Consistent with our meta-analysis, the multi-center PORTEC-3 trial showed that ACR led to better 5-year rates of DFS and OS than adjuvant radiotherapy alone, especially among patients with high-risk types of endometrial cancer [41].

Lymphovascular space involvement (LVSI) and molecular features of endometrial cancer such as those defined in The Cancer Genome Atlas (TCGA) influence prognosis of patients with endometrial cancer and potentially the efficacy of different adjuvant therapies [42, 43]. For example, both the NCCN and the World Health Organization have recommended analyzing TCGA-based molecular features in endometrial cancer patients [8, 44]. Unfortunately, we could not analyze the influence of LVSI or molecular features in our sample because few studies reported relevant data. This should be a focus of future research.

Our meta-analysis presents several limitations. First, pooled OS data showed high heterogeneity, the sources of which we were unable to identify. Nevertheless, our OS meta-analysis seems reliable because we obtained similar results in subgroup analyses, which involved fewer confounding factors. Second, we were unable to take into account additional factors that might affect survival, such as residual tumor volume after surgery, differences in chemo- or radiotherapy regimens, lymph node dissection, LVSI, or TCGA-based molecular characteristics. Third, all but one study in our meta-analysis were retrospective studies, which may increase the risk of selection bias. In addition, we could not exclude data for the 22 patients in stages I, II or IV in the RCT in our meta-analysis. Fourth, the included studies varied in whether they delivered radiotherapy in ACR as external pelvic radiotherapy and/or vaginal brachytherapy, which may have contributed to the observed heterogeneity. Finally, we had to extract HRs from Kaplan–Meier plots in nearly half the studies [17, 26, 27, 31, 34, 35], which may have introduced error.

Despite these limitations, our meta-analysis provides strong evidence that ACR is superior to AC for preventing recurrence and improving survival of patients with stage III endometrial cancer. It may be appropriate to recommend ACR to patients with stage III disease, especially those at greater risk of recurrence.

## Data Availability

The datasets analyzed in the current study are available from the corresponding author on reasonable request.
